# Prenatal Diagnosis of Glutaric Acidemia I Based on Amniotic Fluid Samples in 42 Families Using Genetic and Biochemical Approaches

**DOI:** 10.3389/fgene.2020.00496

**Published:** 2020-05-20

**Authors:** Bing Xiao, Wenjuan Qiu, Jun Ye, Huiwen Zhang, Hong Zhu, Lei Wang, Lili Liang, Feng Xu, Ting Chen, Yan Xu, Yongguo Yu, Xuefan Gu, Lianshu Han

**Affiliations:** ^1^Department of Pediatric Endocrinology and Genetic Metabolism, Institute of Pediatric Research, Xinhua Hospital, School of Medicine, Shanghai Jiaotong University, Shanghai, China; ^2^Center for Prenatal Diagnosis, Xinhua Hospital, School of Medicine, Shanghai Jiao Tong University, Shanghai, China

**Keywords:** glutaric acidemia I, prenatal diagnosis, glutarylcarnitine, glutaric acid, mass spectrometry

## Abstract

Direct mutation analysis is the major method for glutaric acidemia I (GA-I) prenatal diagnosis, while systemic application of a biochemical strategy is rare. We describe our experiences with metabolite measurement together with mutation analysis in GA-I prenatal diagnosis at a single center over 10 years. The data of genetic analysis and metabolite measurement using gas chromatography/mass spectrometry(GC/MS) and tandem mass spectrometry(MS/MS) in amniotic fluid samples of 44 fetuses from 42 GA-I families referred to our center from 2009 to 2019 were retrospectively analyzed. Among these 44 fetuses, genetic and biochemical results were both available in 39 fetuses. Of these, 6 fetuses were judged as affected and 33 fetuses as unaffected by mutation analysis. The levels of glutarylcarnitine (C5DC), C5DC/octanoylcarnitine (C8), and glutaric acid in the supernatant of amniotic fluid from affected fetuses were significantly higher than those in unaffected fetuses [1.73μmol/L (0.89–4.19) vs. 0.16μmol/L (0.06–0.37), 26.26 (12.4–55.55) vs. 2.23 (1.04–8.44), and 103.94 mmol/mol creatinine (30.37–148.31) vs. 1.01mmol/mol creatinine (0–9.81), respectively; all *P* < 0.0001]. Among all families, two were found to have one causative mutation in the proband, in four pregnancies from these two families, three fetuses were judged as “unaffected” and one was judged as “affected” according to metabolites results. Postnatal follow-up showed a normal phenotype in all unaffected fetuses judged by mutation or metabolite analysis. C5DC, C5DC/C8, and glutaric acid levels in the supernatant of amniotic fluid showed significant differences and no overlap between the affected and unaffected fetuses. Biochemical strategy could be implemented as a quick and convenient method for the prenatal diagnosis of GA-I.

## Introduction

Glutaric acidemia I (GA-I) is an autosomal recessive metabolic disorder of lysine, hydroxylysine, and tryptophan metabolism caused by a deficiency of the Glutaryl-CoA Dehydrogenase (GCDH) enzyme (Hedlund et al., 2006). Deficiency of the GCDH enzyme occurs due to pathogenic mutations in the *GCDH* gene mapped at chromosome 19p13.2, which leads to increased organic acid excretion of glutaric acid and 3-hydroxyglutaric acid in urine and elevated glutarylcarnitine (C5DC) in plasma. These metabolites can be reliably detected by gas chromatography/mass spectrometry (GC/MS) and tandem mass spectrometry (MS/MS) in body fluids, such as urine, plasma, cerebrospinal fluid, and body tissues (Baric et al., 1999; Chace et al., 2003).

GA-I is characterized by gliosis and neuronal loss in the basal ganglia and a progressive movement disorder that usually begins during the first year of life (Goodman et al., 1995). Its outcome relies on early diagnosis and treatment. If untreated, 80–90% of infants will develop neurologic disease during the first 6 years of life, which can result in striatal injury and consequent dystonic movement disorder (Boy et al., 2017). Prenatal diagnosis is an important way for the family with GA-I proband to prevent the recurrence of GA-I. Various methods have been reported for the prenatal diagnosis of GA-I, including using a biochemical strategy to measure the glutaric acid level and GCDH activity in amniotic or chorionic villus sampling (Goodman et al., 1980; Christensen, 1994) and genetic analyses to directly screen pathogenic mutations identified in the proband (Busquets et al., 1998, 2000; Lin et al., 2002; Peng et al., 2018). Each method has its own advantages and disadvantages. Measurements of enzyme activity in cultured amniotic cells are time-consuming and troublesome. In addition, it has been reported that some patients have normal excretion of glutarate and high residual GCDH activity (Pineda et al., 1998). Mutation analysis using amniocytes or chorionic villi requires information on the proband, while some probands are found to carry only one causative mutation. In addition, maternal cell contamination may potentially lead to “false negative” results (Hasegawa et al., 2005). Therefore, it is difficult to make a precise prenatal diagnosis based on only one method.

In this study, we describe our experiences in the prenatal diagnosis of GA-I by measurement of metabolites in the supernatant of the amniotic fluid together with direct mutation analysis in amniotic sampling in 44 high-risk pregnancies.

## Methods

### Families and Probands

In this study, there were a total of 42 families (44 pregnancies) with the GA-I proband who sought prenatal diagnosis from January 2009 to December 2019 in our center. The probands were diagnosed based on clinical symptoms, biochemical results obtained by GC/MS and MS/MS, together with a genetic test of the GCDH gene. The mutation spectrum of the *GCDH* gene in this study is shown in Tables 1, 2. Written informed consent was obtained from all participants. Study protocols were approved by the Ethics Committee of Xin Hua Hospital. The study complied with the World Medical Association Declaration of Helsinki regarding the ethical conduct of research involving human subjects.

**Table 1:** Direct sequencing results and three biomarker levels in the amniotic fluid samples of 37 unaffected fetuses.

**Fetal sample number**	**Variants in the proband (NM_000159.4)**		**Fetus status**	**Variant of fetus**	**Metabolites of amniotic fluid**		
	**Allele 1 (paternal)**	**Allele 2 (maternal)**			**C5DC (μmol/L)**	**C5DC/C8**	**Glutaric acid (mmol/mol creatinine)**
F1	c.1244-2A>C	c.1244-2A>C	Carrier	c.1244-2A>C	0.24	2.3	2.15
F2	c.383G>A	c.334+2T>C	Normal	-	0.16	2.4	0
F3	c.784G>A	c.1286C>T	Carrier	c.1286C>T	**0.37**	2.03	0
F4*	ND	c.373C>T	ND	-	0.06	1.07	0
F5	c.532G>A	c.1244-2 A>C	Normal	-	0.09	1.48	0
F6	c.637 ins T	c.145_148delGACT	Carrier	c.145_148delGACT	0.22	**3.88**	1.69
F7	c.767T>C	c.1156C>G	ND	-	0.20	1.29	0
F8	c.383G>A	c.413G>A	Carrier	c.383G>A	0.19	1.28	1.85
F9*	ND	c.373C>T	ND	-	0.10	1.07	1.95
F10	c.1244-2 A>C	c.1244-2 A>C	Carrier	c.1244-2 A>C	0.26	**6.07**	**2.53**
F11	c.91G>T	c.493C>A	Carrier	c.493C>A	0.19	3.00	**2.52**
F12	c.1286C>T	c1244-2A>C	Carrier	c.1286C>T	0.14	2.23	2.02
F13	c.901G>A	c.416G>C	Carrier	c.901G>A	0.15	3.42	**3.11**
F14	c.769C>T	c.1157G>A	Carrier	c.769C>T	0.09	**8.44**	**4.21**
F15	c.263G>A	c.263G>A	Carrier	c.263G>A	0.27	**4.36**	**6.88**
F16	c.1244-2A>C	c.1133C>T	Normal	**-**	**0.31**	**4.21**	**2.93**
F17	c.245G>C	c.1244-2A>C	Normal	-	0.28	**4.17**	0
F18	c.317T>C	c.395G>A	Normal	-	0.13	2.38	0
F19	c.1147C>T	c.647C>T	Normal	**-**	**0.31**	**4.76**	1.24
F20	c.1240G>A	c.1240G>A	Carrier	c.1240G>A	0.19	1.68	0
F21	c.415C>T	c.214G>T	Carrier	c.415C>T	0.17	1.71	0
F22	c.339delT	c.406G>T	Carrier	c.406G>T	0.14	1.12	0.47
F23	c.767T>C	c.1156C>G	Normal	-	0.11	1.13	**9.81**
F24	c.1064G>A	c.1147C>T	Carrier	c.1147C>T	0.10	0.87	**6.99**
F25	c.1064G>A	c.1064G>A	Normal	-	0.25	1.89	0.93
F26	c.1286C>T	c.1244-2 A>C	Carrier	c.1286C>T	0.24	**4.25**	1.23
F27	c.1244-2A>C	c.108_109delAC	Carrier	c.1244_2A>C	0.13	2.28	1.9
F28	c.406G>T	c.881G>A	Carrier	c.406G>T	0.10	2.17	1.01
F29	c.1244-2A>C	c.1244-2A>C	Carrier	c.1244-2A	0.16	1.48	1.66
F30	c.1024G>A	c.395G>A	Carrier	c.395G>A	0.16	1.39	0.89
F31^#^	ND	c.533G>A	ND	-	0.25	3.36	0
F32	c.914C>A	c.1189G>A	Carrier	c.1189G>A	0.16	1.94	0
F33	c.1244-2 A>C	c.1244-2 A>C	Normal	-	0.12	1.27	0
F34	c.892G>A	c.478C>A	Carrier	c.892G>A	0.09	1.76	0
F35	c.1064G>A	c.148T>C	Carrier	c.148T>C	0.11	1.04	0
F36	c.755G>A	c.533G>A	Normal	-	0.30	3.38	0
F37	c.406G>T	c.1169G>A	Carrier	c.406G>T	0.23	3.25	0
Reference range					<0.3	<3.7	<2.5

ND, not determined; *F4 and F9 were two pregnancies from one family; elevated metabolites are shown in bold.

# *F31 and F41 in Table 2 were two pregnancies from one family*.

**Table 2:** Direct sequencing results and three biomarkers levels in the amniotic fluid samples of 7 affected fetuses.

**Fetal sample number**	**Variants in the proband (NM_000159.4)**		**Fetus status**	**Metabolites of amniotic fluid**		
	**Allele 1 (paternal)**	**Allele 2 (maternal)**		**C5DC (μmol/L)**	**C5DC/C8**	**Glutaric acid (mmol/mol creatinine)**
F38	c.413G>A	c.383G>A	Affected	1.54	12.40	90.09
F39	c.1240G>A	c.1240G>A	Affected	0.89	17.20	111.61
F40	c.263G>A	c.263G>A	Affected	1.63	25.93	148.31
F41^**#**^	ND	c.533G>A	ND	2.85	22.28	266.02
F42	c.797T>C	c.1064G>A	Affected	4.19	55.56	122.94
F43	c.1235C>A	c.1244-2A>C	Affected	1.84	26.59	30.37
F44	c.532G>A	c.356C>T	Affected	3.34	29.46	96.27
Reference range				<0.3	<3.7	<2.5

ND, not determined;

# *F41 and F31 in Table 1 were two pregnancies from one family*.

### Amniocyte Samples

Amniocentesis was performed by experienced obstetricians at gestational ages ranging from 16 to 20 weeks. In each case, 30 ml of amniotic fluid was obtained from each pregnant woman, 10 ml amniotic fluid was used for DNA extraction, and supernatant samples were used for the metabolite analysis. The residual 20 ml of amniotic fluid was cultured in a flask for karyotyping analysis. Further, 3–4 ml of peripheral blood was collected from all GA-I pedigree members to perform a linkage analysis and to exclude maternal cell contamination.

### Metabolite Test and Analysis

Informative markers, and common informative ratios for GA disorders have been selected according to guideline issued by CLSI (2010). The levels of C5DC and octanoylcarnitine (C8) were quantitatively analyzed by MS/MS (Applied Biosystems, API 2000) (Han et al., 2007) using 3 μl uncultured amniotic fluid supernatant samples and then the levels were automatically calculated based on the assigned values for internal standards using ChemoView v1.2 software. Quality control samples were provided by the Centers for Disease Control and Prevention (Atlanta, GA, USA). The organic acid fraction was extracted, methylated, and analyzed by chemical ionization GC/MS (GC-MS-QP2010; Shimadzu Limited, Kyoto, Japan) operated in selected ion monitoring mode. For each amniotic fluid supernatant sample, 2 ml uncultured amniotic fluid supernatant samples was mixed with stable isotope-labeled compounds and internal standards as described by Hasegawa et al. (2005). Concentrations of glutaric acid in the amniotic fluid were calculated using GC-MS Solution v2.40 software.

### Direct DNA Mutation Screening by Sanger Sequencing

Genomic DNA was extracted from 10 ml uncultured amniotic fluid using a QIAamp DNA Blood Mini Kit (Qiagen Inc., Valencia, CA, USA), and the candidate variants were detected using Sanger sequencing. PCR primer sequences and protocols are available upon request. Amplified fragments were sequenced using a 96-capillary 3730xl system (Applied Biosystems).

### Linkage Analysis and Exclusion of Maternal Material Contamination

Seven closely linked flanking short tandem repeat (STR) markers at the *GCDH* gene locus were selected to perform the linkage analysis and to exclude maternal cell contamination. (STR markers and primers are listed in Supplementary Table 1).

### Statistical Analysis

Standard scatter plots were generated for C5DC, C8, and glutaric acid levels in each fetus by affected and unaffected groups. Wilcoxon rank sum tests with exact p values were performed to compare the C5DC, C8, and glutaric acid levels in affected and unaffected groups.

## Results

The genetic and biochemical results for the 44 fetal samples from 42 families are listed in Tables 1, 2. Among the 44 fetuses, 7 fetuses were judged as “affected” and 37 fetuses were judged as “unaffected” by the metabolite test for the supernatant of the amniotic fluid or the mutation analysis of amniocyte samples.

Seven fetuses showing higher levels of C5DC, C5DC/C8, and glutaric acid (Table 2) compared to those in controls were judged as “affected,” among which 6 fetuses showed homozygous or compound heterogeneous mutations in the *GCDH* gene by direct sequencing. In this group, the median levels of C5DC, C5DC/C8, and glutaric acid in the amniotic fluid were 1.73 μmol/L (0.89–4.19), 26.26 (12.4–55.55), and 103.94 mmol/mol creatinine (30.37–148.31), respectively. Another fetus (F41) showed higher levels of C5DC (2.85 μmol/L), C5DC/C8 (22.28), and glutaric acid (266.02 mmol/mol creatinine), while only one causative variant was found in the proband of the family; the genetic test could not make a precise prenatal diagnosis for this family. According to the levels of C5DC, C5DC/C8, and glutaric acid in the supernatant of amniotic fluid, this fetus was judged as “affected”; the parents chose to terminate the pregnancy.

A total of 37 fetuses were judged as “unaffected” by the metabolite test for the supernatant of the amniotic fluid or the mutation analysis of amniocyte samples. Among them, 33 fetuses carried one heterogeneous mutation (*n* = 23) or no mutation (*n* = 10) in the *GCDH*, and the median levels of C5DC, C5DC/C8, and glutaric acid in the amniotic fluid were 0.16 μmol/L (0.06–0.37), 2.23 (1.04–8.44), and 1.01 mmol/mol creatinine (0–9.81), respectively. Another three fetuses (F4, F9, and F31) from two families were found to have one causative mutation in the proband, and the C5DC, C5DC/C8, and glutaric acid levels were in the normal range (Table 1); all of them were judged as “unaffected.” Another fetus sample (F7) was found to have visible maternal cell contamination, and the C5DC, C5DC/C8, and glutaric acid levels in the amniotic fluid were 0.20 μmol/L, 1.29, and 0 mmol/mol creatinine, respectively; this fetus was judged as “unaffected.” Postnatal follow-up of all these 37 fetuses showed a normal phenotype.

In the 33 unaffected fetuses judged by mutation analysis, a discrepancy among three metabolites C5DC, C5DC/C8, and glutaric acid levels in several amniotic fluid samples was observed (Table 1). The C5DC level in F3 was slightly higher than the normal range, while the levels of the other two metabolites were in the normal range; three samples showed slightly elevated C5DC/C8; four samples showed elevated glutaric acid level; one sample showed elevated C5DC and C5DC/C8 and normal glutaric acid; three samples showed elevated C5DC/C8 and glutaric acid and normal C5DC; and one sample showed slightly elevated C5DC, C5DC/C8, and glutaric acid levels.

In addition, in the *GCDH* mutation carrier group (*n* = 23), the median levels of C5DC, C5DC/C8, and glutaric acid in the amniotic fluid were 0.16 μmol/L, 2.17, and 1.66 mmol/mol creatinine, respectively. In the *GCDH* mutation normal fetus group (*n* = 10), the median levels of C5DC, C5DC/C8, and glutaric acid in the amniotic fluid were 0.206 μmol/L, 2.392, and 0 mmol/mol creatinine, respectively. The results showed no significant difference between these two groups (*P* > 0.05). Therefore, no relationships between genotype and levels of metabolites in the amniotic fluid were observed.

**Figure 1:**
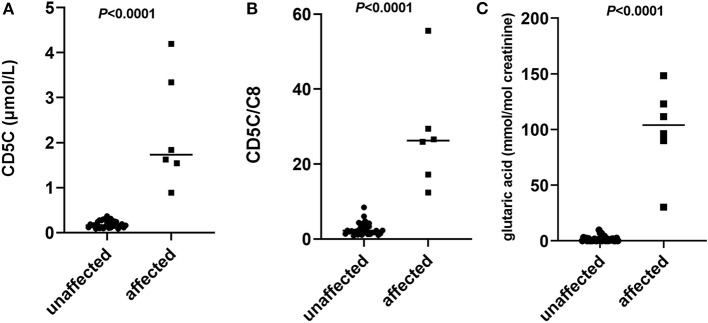
Scatter plots of individual levels of glutarylcarnitine (C5DC) **(A)**, C5DC/octanoylcarnitine (C8) **(B)**, and glutaric acid **(C)** in the supernatant of amniotic fluid of 33 affected fetuses and 6 unaffected fetuses judged by mutation analysis. Horizontal lines, median values. *P*-values were determined by the Wilcoxon rank sum test.

According to the results in Tables 1, 2, C5DC, C5DC/C8, and glutaric acid levels in the supernatant of amniotic fluid showed significant differences (*P* < 0.0001), and there was no overlap between the affected and unaffected fetuses judged by the mutation test (Figure 1). The sensitivities of C5DC, C5DC/C8, and glutaric acid were 100%, and the specificities of C5DC, C5DC/C8, and glutaric acid were 91, 76, and 76%, respectively.

Of all the patients who sought GA-I prenatal diagnosis in this study, two probands from two families were found to have only one causative variant. Biochemical tests of two pregnancies (F4 and F9) from one family showed that C5DC, C5DC/C8, and glutaric acid levels were in the normal range, and both pregnancies were judged as “unaffected” and continued; postnatal follow-up showed a normal phenotype. In another family, C5DC, C5DC/C8, and glutaric acid levels of one pregnancy (F41) were significantly higher than those in control and was judged as “affected”; thus, the parents chose to terminate the pregnancy. In another pregnancy (F31), C5DC, C5DC/C8, and glutaric acid levels were in the normal range, and the family decided to continue the pregnancy; postnatal follow-up showed a normal phenotype.

## Discussion

GA-I is a rare inherited metabolic disease with an estimated worldwide incidence of 1:492,000–1:69,165 infants (Frazier et al., 2006; Kölker et al., 2007; Kasper et al., 2010; Naoaki et al., 2018). Most untreated GA-I patients will have poor outcomes, leading to significant mortality and morbidity results. Prenatal diagnosis is an important way for the family with GA-I probands to prevent the recurrence of GA-I. Direct mutation analysis is the major method for the prenatal diagnosis of GA-I. Using a biochemical strategy to measure the glutaric acid level and GCDH activity in amniotic or chorionic villus sampling has been reported, and most of the published articles are case reports or small series (Goodman et al., 1980; Christensen, 1994). Since the description of rare patients with normal or only minimally elevated levels of glutaric acid and high residual GCDH activity, and plasma C5DC can be normal in patients with GA type I without elevation of urine glutaric acid (Lindner et al., 2004; Gallagher et al., 2005), it seems that it may be difficult to make a precise prenatal diagnosis based on these diagnostic metabolites in some rare patients. Therefore, systemic application and evaluation of these diagnostic metabolites in prenatal samples of GA-I are critical. In this study, we used two methods GC/MS and MS/MS simultaneously to measure the metabolites in the supernatant of the amniotic fluid for prenatal diagnosis of 44 high-risk pregnancies.

In this study, from the data of our systematic evaluation of 6 affected fetuses and 33 unaffected fetuses judged by direct mutation analysis, all of the three biochemical markers (CD5DC, CD5DC/C8, and glutaric acid levels) in the supernatant of amniotic fluid showed no overlap between the affected and unaffected fetuses. No discrepancy among the three metabolites was observed in the 6 affected fetuses judged by mutation analysis, which showed high sensitivity of these three makers individually or in combination. However, a discrepancy among the three metabolites was seen in several normal fetuses, which leads to unable clarification between affected and unaffected cases by one or two metabolite levels. One of likely reason might be associated with the selection of the cutoff value. Traditionally, this is defined as a given percentile of the normal population or by adding multiples of the standard deviation to the mean value. It has been suggested that cutoffs selected exclusively based on normal results might result in much false positives (McHugh et al., 2011). The high target range in this study is defined by 99.5th percentile of the accumulative normal fetus. According to this normal range, several normal fetus found with one or two metabolites are outside of this defined range which would be determined as “affected.” Although we can see that the range between affected and unaffected group showed no overlap for these three metabolites. It has been suggested that clinically relevant cutoff target ranges may need to be adjusted in response to the degree of overlap between normal population and disorder range (McHugh et al., 2011). However, due to the small number of the affected group in this study, it is difficult to establish more reasonable ranges in this manner. Therefore, if one or two of these metabolites were higher than the normal range in this study, it is difficult to make a precise prenatal diagnosis relying only on one or two metabolite measurement. These results suggested that C5DC, C5DC/C8, and glutaric acid could be used in combination or together with the genetic test to confirm the results of sequencing in prenatal diagnosis as an independent step to ensure accuracy. Due to the limited sample size of this study, further data are warranted to prove the reliability of these three biomarkers in the prenatal diagnosis and to establish more reasonable target range.

Mutation analysis using amniocytes or chorionic villi was regarded as the most reliable strategy for prenatal diagnosis of GA-I; however, it requires information on the proband, and maternal cell contamination may potentially lead to “false negative” results (Hasegawa et al., 2005). In some patients with GA-I disease, only one causative mutation was found in the proband, like two families in our study; in four pregnancies of these two families, only the genetic test could not make a precise prenatal diagnosis. In such a situation, the metabolite test for the supernatant of the amniotic fluid provides fast and reliable results using a small amount of sample, which can help these families in making a decision. This advantage was also observed in our previous report on prenatal metabolite tests in methylmalonic acidemia (Ji et al., 2019). Another limitation of the mutation analysis potentially leading to “false negative” results is maternal cell contamination. In this study, the amniotic fluid sample of one fetus (F7) showed visible maternal blood contamination; mutation analysis showed that the fetus carried a maternal mutation and the metabolite test showed that the C5DC, C5DC/C8, and glutaric acid levels in the amniotic fluid were in the normal range; the fetus was judged as “unaffected” according to the metabolite test. Postnatal follow-up showed a normal phenotype. Therefore, despite the accuracy of molecular tests for prenatal diagnosis of GA-I, biochemical analysis has an important role in the prenatal diagnosis of families with inconclusive genetic results or maternal cell contamination.

In summary, a biochemical strategy provides an option in the prenatal diagnosis of GA-I, especially in families with inconclusive genetic results. This method is simple, fast, and costless ($70 vs. $200 per test for mutation analysis), and it needs only a small amount of amniotic fluid.

## Data Availability Statement

The datasets generated for this study are available on request to the corresponding author.

## Ethics Statement

The studies involving human participants were reviewed and approved by Ethics Committee of Xin Hua Hospital. The patients/participants provided their written informed consent to participate in this study.

## Author Contributions

LH conceived and designed the study. BX drafted the manuscript. WQ, HZha, LL, XG, YY, LH, and JY enrolled the patients. HZhu and LW performed amniocentesis. FX and TC performed the metabolite test and analysis. YX performed the mutation test. BX participated in the data analysis. All the authors reviewed and approved this submission.

## Conflict of Interest

The authors declare that the research was conducted in the absence of any commercial or financial relationships that could be construed as a potential conflict of interest.
