# Supplementary material for: Prenatal Diagnosis of Glutaric Acidemia I Based on Amniotic Fluid Samples in 42 Families Using Genetic and Biochemical Approaches
Source: Front Genet. 2020 May 20;11:496. doi: 10.3389/fgene.2020.00496 (PMC7251148; doi:10.3389/fgene.2020.00496)
Supplement: Supplementary file 1 [file Table_1.DOCX]

| **STR locus** | **sense primer（5'-3'）** | **antisense primer（5'-3'）** | **PCR annealing temperature** |
| --- | --- | --- | --- |
| D19S906 | ATAGGGTCTGGCCAGGCG | CGGACAGTCCCTGTTCCTTC | 58 |
| D19S1165 | AAGCTATGATGGGTGCCAAT | ATCACTCTTCATTATGGCTTCA | 48 |
| D19S221 | GCAAGACTCTGACTCAACAAAA | CATAGAGATCAATGGCATGAAA | 48 |
| D19S914 | TTGGGACACACAATCCT | TTTCTTGGTGGAGTGGTT | 48 |
| D19S558 | GATTTTCTATTGCATTCAGCT | GACCCTGTCTCAAACAAAC | 48 |
| D19S1150 | GGAGAAGCATAGAAAAGCCA | CCTGTTGAAAACTCCTGACC | 48 |
| D19S840 | ATAGGCCAAGACTGTCTAAAACAA | GCCCTAACTGCTGTAAGAGAACT | 53 |

**Supplementary table 1. Seven closely linked flanking STR markers at *GCDH* gene locus**
